# Mucin 1 Inhibits Ferroptosis and Sensitizes Vitamin E to Alleviate Sepsis-Induced Acute Lung Injury through GSK3*β*/Keap1-Nrf2-GPX4 Pathway

**DOI:** 10.1155/2022/2405943

**Published:** 2022-07-21

**Authors:** Yu-Ming Wang, Fang-Chen Gong, Xing Qi, Yan-Jun Zheng, Xiang-Tao Zheng, Ying Chen, Zhi-Tao Yang, Qing-Ye, En-Qiang Mao, Er-Zhen Chen

**Affiliations:** ^1^Department of Emergency, Ruijin Hospital, Shanghai Jiao Tong University School of Medicine, Shanghai, China; ^2^Department of Thoracic Surgery, Renji Hospital, Shanghai Jiao Tong University School of Medicine, Shanghai, China; ^3^Department of Critical Care Medicine, Shanghai Tenth People's Hospital, Tongji University School of Medicine, Shanghai, China

## Abstract

**Background:**

Ferroptosis is a nonapoptotic form of programmed cell death, which may be related to the occurrence and development of sepsis-induced acute respiratory distress syndrome (ARDS)/acute lung injury (ALI). Mucin 1 (MUC1) is a kind of macromolecule transmembrane glycoprotein. Previous studies have shown that MUC1 could relieve ALI in sepsis and predict whether sepsis patients would develop into ARDS. However, the role of MUC1 in the ferroptosis of sepsis-induced ALI/ARDS remains unclear.

**Materials and Methods:**

Sera samples from 50 patients with sepsis/septic shock were used to detect iron metabolism-related markers. Western blot and qRT-PCR were conducted to detect the expression levels of ferroptosis-related genes. Enzyme-linked immunosorbent assay (ELISA) was performed to evaluate inflammatory factors. Transmission electron microscopy (TEM) was used to assess morphological changes of cells.

**Results:**

The results showed that the iron metabolism-related indicators in sepsis-induced ARDS patients changed significantly, suggesting the iron metabolism disorder. The expression levels of ferroptosis-related genes in lung tissues of sepsis had marked changes, and the lipid peroxidation levels increased, while Ferrostatin-1 (Fer-1) could reverse the above results, which confirmed the occurrence of ferroptosis. In terms of mechanism studies, inhibition of MUC1 dimerization could increase the expression level of Keap1, reduce the phosphorylation level of GSK3*β*, inhibit the entry of Nrf2 into the nucleus, further inhibit the expression level of GPX4, enhance the lipid peroxidation level of lung tissues, trigger ferroptosis, and aggravate lung injury. Besides, inhibiting MUC1 reversed the alleviating effect of vitamin E on ALI caused by sepsis, increased the aggregation of inflammatory cells in lung tissues, and aggravated alveolar injury and edema.

**Conclusions:**

Our study was the first to explore the changes of iron metabolism indicators in ALI/ARDS of sepsis, clarify the important role of ferroptosis in ALI/ARDS induced by sepsis, and reveal the effects and specific mechanisms of MUC1 in regulating ferroptosis, as well as the sensitization on vitamin E.

## 1. Background

Sepsis is a severe clinical syndrome, which is caused by inflammatory reaction dysregulation and subsequent multiple organ dysfunction [1, 2]. Sepsis has high morbidity and mortality [3–5]; with the speeding up of the aging population, the number of patients is on the increase [6, 7]. Severe sepsis or septic shock can lead to functional impairment of various organs, among which the lung is one of the most common and vulnerable organs [8]. It has been reported that about 40% of the occurrence of ARDS/ALI was caused by sepsis [9].

ARDS is a kind of acute diffuse inflammatory injury caused by multiple factors inside and outside the lung [10, 11]. As one of the main causes of acute respiratory failure, ARDS is characterized by increased pulmonary endothelial and epithelial permeability and diffused alveolar damage [12]. Subsequently, a large number of inflammatory factors and reactive oxygen species (ROS) lead to imbalance between oxidative and antioxidant systems, which results in a series of inflammatory responses [13, 14]. Studies have shown that for patients with sepsis-induced ARDS, the progression of the disease is much faster than expected [15, 16]. Currently, there is no specific treatment for sepsis-induced ARDS [8, 17]; therefore, exploring its mechanism is conducive to finding biomarkers for the early identification of sepsis that may develop into ARDS and identifying potential therapeutic targets.

Ferroptosis is a nonapoptotic form of programmed cell death, which was first discovered in 2012 [18, 19]. Different from other kinds of programmed cell death, ferroptosis is characterized by iron dependence and accumulation of intracellular lipid peroxides [18], and a variety of molecular signaling pathways is involved [20]. In addition, ferroptosis has its unique morphological and bioenergetic characteristics, mainly including the reduction or disappearance of the mitochondrial crest, increase of membrane density, and destruction of membrane integrity [21]. Many studies have shown that ferroptosis is closely related to damage-associated molecular patterns (DAMPs) and sustained release of cytokines, which further promote the intensification of the inflammatory response. Therefore, ferroptosis is considered as an immune-derived cell death [22, 23]. The release of inflammatory cytokines further accelerates the occurrence of ferroptosis and other forms of cell death, thus forming a cascade amplification effect and exacerbating damage of organs [24]. Relevant studies have reported that the imbalance of oxides and antioxidants played an important role in sepsis-induced ALI/ARDS [25–27]; however, the specific mechanism needed to be elucidated. In this study, we aim to clarify the correlation between ferroptosis and the occurrence, development, and prognosis of ALI/ARDS in sepsis.

MUC1 is a high molecular weight type I transmembrane glycoprotein, mainly composed of MUC1-N and MUC1-C, two noncovalently bound polypeptides [28, 29]. The primary role of MUC1-N is antiadhesion and lubrication, and MUC1-C is mainly related to the activation of various signal pathways [30]. In recent years, many studies have shown that MUC1 had effective anti-inflammatory functions, which were mainly concentrated in the respiratory system [31, 32]. Our previous studies found that MUC1 played an important role in the inhibition of the TLR-4-NF-*κ*B inflammatory pathway by paclitaxel in alleviating acute lung injury of septic mice [33]. Meanwhile, we also found that the expression level of MUC1 (KL-6) in the plasma of sepsis patients complicated with ARDS was significantly increased and had a good predictive value for early sepsis patients complicated with ARDS [34]. Li et al. found that MUC1 was a ferroptosis-related gene and had an important prognostic value in idiopathic pulmonary fibrosis [35]. Cui et al. identified MUC1 as a possible marker of ferroptosis in ulcerative colitis through database integration analysis [36]. However, whether MUC1 plays a role in the ferroptosis of ALI/ARDS in sepsis remains obscure, and the specific mechanism needs to be illustrated.

Vitamin E, as a lipophilic antioxidant, could reduce or terminate the damaging chain reaction caused by ROS to alleviate ferroptosis [37]. Clinical benefits of vitamin E include regulation of oxidative stress, inflammation, and cell death. It has been reported that vitamin E regulated ferroptosis through the regulatory mechanism of oxidoreductase 15-lipooxygenase (15-LO), but the mechanism remained unclear [38, 39]. Other studies have found that vitamin E could act synergistically with GPX4 to prevent lipid peroxidation [40]. Currently, the role and mechanism of vitamin E in ferroptosis from ALI/ARDS in sepsis has not been elucidated, and there is a lack of relevant studies on the interaction between MUC1 and vitamin E.

In this study, we first tested iron metabolism-related indicators from the sera of sepsis/septic shock patients and found that there were marked iron metabolism disorders. Through in vivo and in vitro model of sepsis-induced ALI, expression levels of ferroptosis-related genes, and oxidation reduction product levels of lung tissues had obvious changes, which prompted the occurrence of ferroptosis. Further research suggested that MUC1 might alleviate lung injury by inhibiting ferroptosis and have a sensitization effect on vitamin E, and the corresponding mechanism was revealed for the first time.

## 2. Materials and Methods

### 2.1. Samples of Enrolled Patients

50 patients with sepsis or septic shock were enrolled. Relevant clinical data have been analyzed in our previous study [34]. This study was approved by the Ethics Committee of Ruijin Hospital (No. 20210101). The investigation was based on the institution's guidelines for human studies and conformed to the ethics guidelines of the Declaration of Helsinki.

Inclusion criteria are as follows: 18 years old < age < 90 years old, meet the diagnostic criteria of sepsis 3.0 for sepsis/septic shock, and stay longer than 24 hours; patients who did not develop sepsis or septic shock at the time of admission but developed the condition during hospitalization were excluded.

Exclusion criteria were as follows: patients discharged from hospital or died within 24 hours after admission, patients who participated in other clinical studies, patients who required emergency surgery upon admission, patients suffering from a malignant tumor, pregnant or breast-feeding patients, and patients lacking necessary clinical data.

Patients were divided into the ARDS group and No ARDS group according to the occurrence of ARDS during hospitalization and the survival group and nonsurvival group according to the survival of patients. Blood samples were collected from all enrolled patients on the day of admission to detect the levels of sera iron, ferritin, transferrin, and transferrin saturation, and 30 healthy adults were regarded as controls.

### 2.2. Animals

C57BL/6J male mice (6–8 weeks) were purchased from Slac Lab Animals (Shanghai, China) and kept in a temperature-regulated (21–23°C) and humidity-regulated (30–70%) room with a 12-hour light/dark cycle. There was no dietary or water restrictions for the animals. All animal experiments were in accordance with guidelines from the Ruijin Hospital Ethical Committee of Shanghai Jiao Tong University School of Medicine (No. 092) and the International Guide for the Care and Use of Laboratory Animals (National Academy of Sciences Health Publication No. 85–23, revised in 1996).

### 2.3. Reagents and Group

MUC1 inhibitor GO203 (Selleck Chemicals, Houston, TX, USA) and its negative control CP2 (Selleck Chemicals, Houston, TX, USA) were used for in vivo and in vitro experiments. For in vivo experiments, GO203 or CP2 (20 mg/kg) was dissolved in PBS and injected intraperitoneally (*n* = 6 for each group). For in vitro experiments, cells were treated with 5 *μ*M GO203 or CP2 for 48 h. Ferrostatin-1 (Fer-1), CT99021, and vitamin E were all purchased from Selleck Chemicals and used for in vivo or in vitro studies, the efficiency of which was detected via western blot. For in vivo experiments, Fer-1 (5 mg/kg, dissolved in PBS) was injected intraperitoneally 1 h after CLP modeling. 2.5 mg/kg of vitamin E was injected intraperitoneally twice per day (*n* = 6 for each group). For in vitro experiments, the dosage of Fer-1 was 2 *μ*M, the dosage of CT99021 was 2 nM, and the dosage of vitamin E was 10 *μ*M.

### 2.4. CLP Model

The mice model of sepsis was constructed using the Cecal Ligation and Puncture (CLP) method. C57BL/6 male mice (6-8 weeks) were used as the research objects. The basic principle of the CLP method was to find the caecum through anatomy and puncture at the blind end and extrude the contents into the abdominal cavity. Diffuse peritonitis was formed, and systemic infection appeared in mice. Mice in the control group were only treated with laparotomy. All mice were sacrificed 24 h after modeling, and 20-30 g of lung tissues was placed into cryopreserved tubes and stored at -80°C. Sera and bronchoalveolar lavage fluid (BALF) were also taken for subsequent experiments.

### 2.5. Cell Culture

We purchased the murine lung epithelial cell line (MLE-12) from the Institute of Stem Cell Research within the Chinese Academy of Sciences (Shanghai, China). MLE-12 cells were cultured in DMEM-f12 (Gibco, Grand Island, NY, USA) supplemented with 10% heat-inactivated fetal calf sera (Gibco), 1% penicillin, and streptomycin (Millipore, Waltham, MA, USA) in a 37°C incubator with 5% CO_2_.

### 2.6. Histological and Biochemical Analysis

We first fixed lung tissues in 4% paraformaldehyde for 24 h and then stained them using hematoxylin and eosin staining (HE staining). The severity of organ injury was assessed on the basis of the criteria [41, 42]. At least six areas of view at 100x magnification were double-blind evaluated by three pathologists.

The lung tissues were weighed (wet weight) at 24 h after modeling and then placed in the oven at 60°C for 24 h and weighed again (dry weight) to calculate the wet-dry weight ratio. Student's *t* test was used to evaluate differences between groups.

### 2.7. Enzyme-Linked Immunosorbent Assay (ELISA)

The changes of TNF-*α*, IL-1*β*, IL-6, and IL-10 in sera and BALF of mice (*n* = 6 for each group) were detected using an ELISA Kit (MultiSciences Biotechnology Co., Ltd., Hangzhou, China), and all programs were in accordance with manufacturer's protocol. Student's *t* test was used to evaluate differences between groups.

### 2.8. qRT-PCR

We first used a TRIzol reagent (Invitrogen, Carlsbad, CA, USA) to extract total RNA from lung tissues and cell lines, then carried out reverse transcription using the HiScript III RT SuperMix (Vazyme, Nanjing, China), and finally used the AceQ Universal SYBR qPCR Master Mix (Vazyme, Nanjing, China) to detect total RNA under recommended conditions. GAPDH was used as the negative control. Sequences of primers were synthesized by Bioegene Co., Ltd. (Shanghai) and listed in Table S1. Sequences of Keap1 siRNA were as follows: NC sense 5′UUCUCCGAACGUGUCACGUdTdT3′, NC antisense 5′ACGUGACACGUUCGGAGAAdTdT3′, Keap1 siRNA sense 5′GUGGCGAAUGAUCACAGCAAUTT3′, and antisense 5′AUUGCUGUGAUCAUUCGCCACTT3′. We set three independent events for each group.

### 2.9. Western Blot

Protein samples from cells or lung tissues were lysed using the RIPA buffer, then subjected to 10% SDS-PAGE and transferred onto polyvinylidene fluoride (PVDF) membranes. Primary antibodies including anti-ASCL4, anti-SLC7A11, anti-ATF4, anti-GPX4, anti-Nrf2, anti-Keap1, anti- GSK3*β*, and anti-pGSK3*β* were used (Table S2). GAPDH and histone H3 were used as the negative control.

### 2.10. Nuclear and Cytoplasmic Extraction

The cytoplasmic and nuclear proteins were purified using NE-PER Nuclear and Cytoplasmic Extraction Reagents (Pierce Biotechnology, Inc., Rockford, IL, USA) according to the manufacturer's protocol. Western blot was conducted to detect expression levels of cytoplasmic and nuclear proteins.

### 2.11. Transmission Electron Microscopy

The adherent cell samples were first removed from the culture medium and fixed with 2.5% glutaraldehyde for 5 min. Cells were gently removed with cell scraping, transferred into the centrifuge tube, and centrifuged at 3000 rpm for 2 min. The fixator was removed, and a new electron microscope fixator was added. Then, cells were dehydrated with ethanol, embedded with pure acetone+embedding solution (2 : 1), cured, sliced with an ultrathin slicer, stained, and photographed with transmission electron microscopy.

### 2.12. Iron Metabolism Indicator Measurement

Iron and Fe^2+^ concentrations in sera, BALF, and lung tissues were detected using the Iron Assay Kit (MAK025-1KT, Sigma) according to the manufacturer's instructions. Total iron and Fe^2+^ were measured in 10 *μ*L of sera and BALF or 10 mg of tissue homogenate. Absorption was measured at 593 nm and compared with a standard curve for known concentrations.

Ferritin, transferrin, and transferrin saturation from sera of sepsis or septic shock patients were measured in accordance with the manufacturer's protocol (Servicebio Technology, Wuhan, China). Student's *t* test was used to evaluate differences between groups.

### 2.13. Reactive Oxidative Stress Activity Assay

The lung tissues were weighed accurately. Precooled normal saline was added according to the weight-to-volume ratio, and the lung tissues were ground at high speed. The levels of redox products (glutathione (GSH), malondialdehyde (MDA), myeloperoxidase (MPO), and superoxide dismutase (SOD)) in the lung tissues were detected using commercial biochemical kits (Nanjing Jiancheng, China) following the manufacturer's instructions. Student's *t* test was used to evaluate differences between groups.

### 2.14. Lipid Peroxidation Activity Assay

To determine the level of lipid peroxides, C11 BODIPY 581/591 (50 *μ*M) was added to the treated cells and incubated for 1 h. The cells were cleaned with PBS to remove the excess dye, then digested with trypsin, and resuspended in PBS (containing 5% FBS) for flow cytometry analysis. We set three independent events for each group.

### 2.15. Statistical Analysis

SPSS 20.0 (IBM, SPSS, Chicago, IL, USA) and GraphPad Prism 7.0 (GraphPad Software Inc., CA, USA) were used to analyze data which were expressed as mean ± standard deviation (SD). Student's *t* test and one-way ANOVA were used to evaluate differences between groups. *P* value < 0.05 was considered statistically significant.

## 3. Results

### 3.1. Changes of Iron Metabolism-Related Indicators in Sepsis-Induced ALI/ARDS

**Figure 1:**
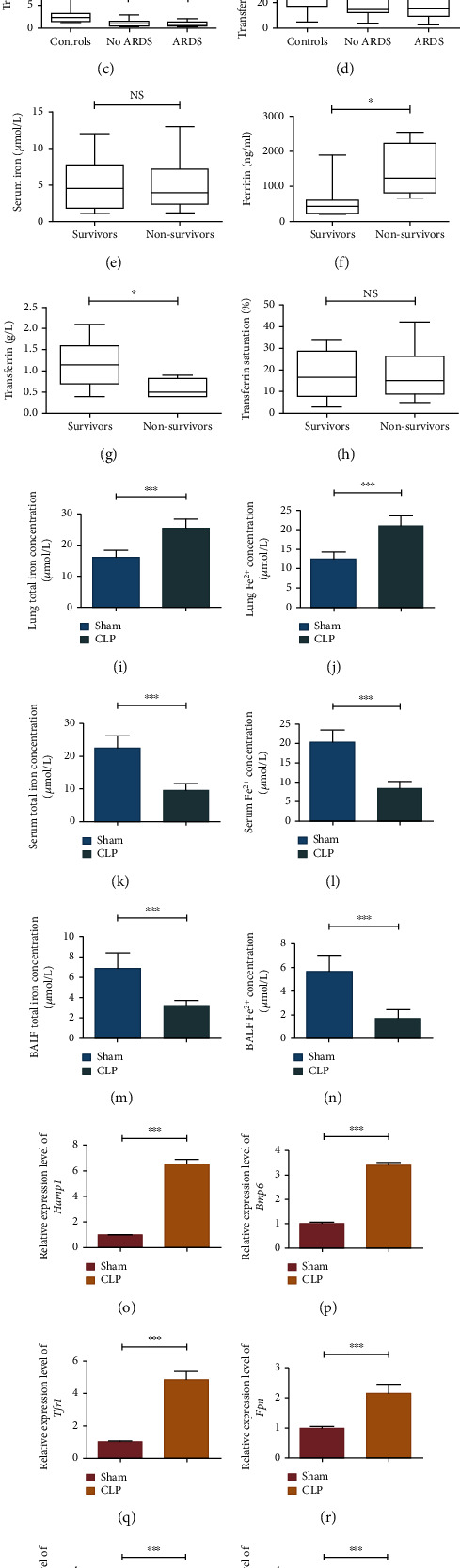
Changes of iron metabolism-related indicators in sepsis-induced ALI/ARDS. (a–d) Differences of iron metabolism-related indicators in sepsis patients complicated with or without ARDS; (e–h) differences of iron metabolism-related indicators between survival group and nonsurvival group in sepsis patients complicated with ARDS; (i–n) expression levels of total iron and divalent iron in lung tissues, sera, and BALF of sepsis mice; (o–t) expression levels of iron metabolism-related genes in lung tissues of sepsis mice. All data were expressed in the form of mean ± standard deviation; ^∗^*P* < 0.05, ^∗∗^*P* < 0.01, and ^∗∗∗^*P* < 0.001.

We collected data from 50 patients with sepsis/septic shock, including 34 patients without ARDS, 16 patients with ARDS, and another 30 healthy controls. By detecting serum total iron, ferritin, transferrin, and transferrin saturation levels of all the patients during the first day in the hospital, we found that ferritin of sepsis patients was higher than that of the control group, while total iron, transferrin, and transferrin saturation were lower in the control group, however, whether or not sepsis patients with or without ARDS had an significant effect on sera iron metabolism indicators (Figures 1(a)–1(d)). Further, patients with sepsis complicated with ARDS were divided into the survival group (*n* = 10) and nonsurvival group (*n* = 6). Results showed that ferritin in the nonsurvival group was significantly higher than that in the survival group, but transferrin was significantly lower in the survival group, with no significant differences in other indicators (Figures 1(e)–1(h)). In animal experiments, the CLP method was used to construct the sepsis mouse model, and the levels of total iron and divalent iron in lung tissues, sera, and bronchoalveolar lavage fluid (BALF) of sepsis and control mice were detected, respectively. Results showed that the levels of total iron and divalent iron in lung tissues of sepsis mice were significantly higher than those of the control group (Figures 1(i) and 1(j)), while the levels of total iron and divalent iron in sera and BALF were lower in the control group (Figures 1(k)–1(n)). Expression levels of iron metabolism-related genes including Hamp1, Bmp6, Tfr1, Fpn, Fth, and Ftl in lung tissues were detected using qRT-PCR, and the results indicated that expression levels of the above genes in the CLP group were significantly higher than those in the control group (Figures 1(o)–1(t)).

### 3.2. Ferroptosis Occurred in the Lung of Septic Mice

**Figure 2:**
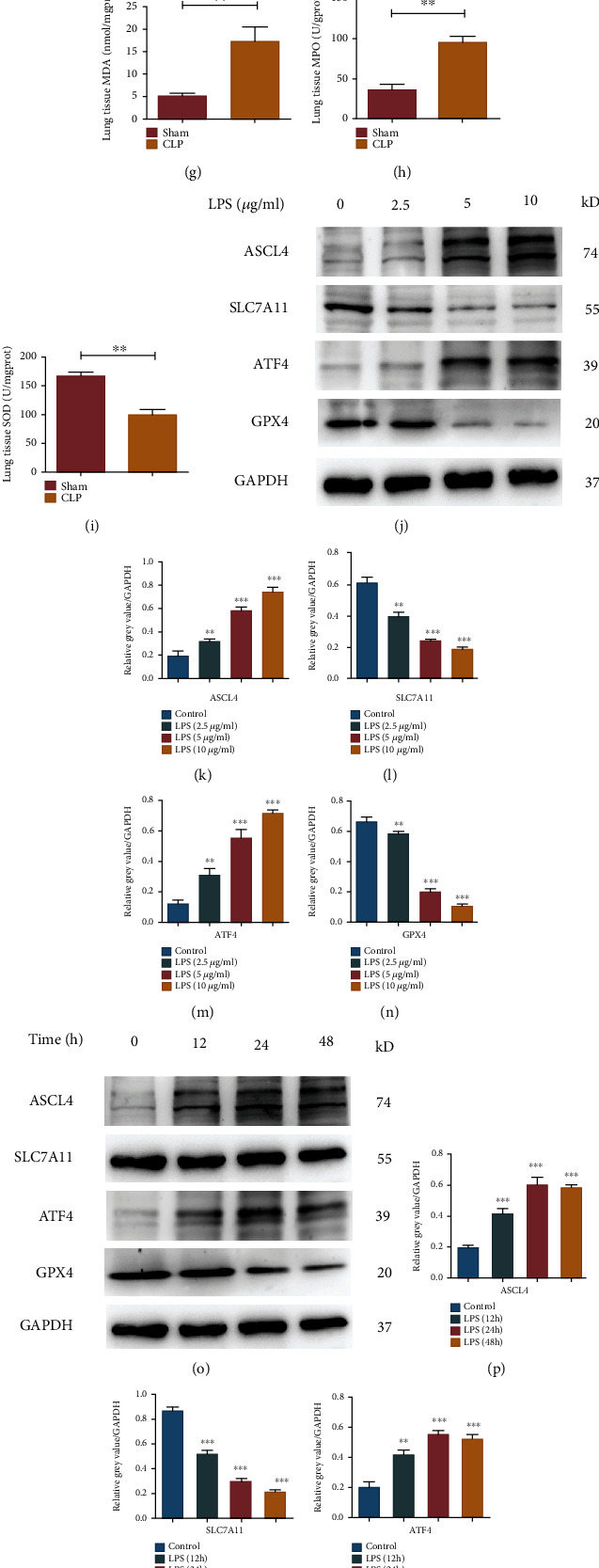
Ferroptosis occurred in lung tissues and alveolar epithelial cells of sepsis. (a–e) Western blot assay was used to evaluate the expression levels of ferroptosis-related genes; (f–i) expression levels of redox products in the lung tissues of sepsis mice; (j–n) effects of different concentrations of LPS on the expression levels of ferroptosis-related genes; (o–s) effects of different time points of LPS on the expression levels of ferroptosis-related genes; (t, u) flow cytometry was used to detect the levels of lipid peroxides in MLE-12 cells; (v) TEM assessment of the effects of LPS on ferroptosis-related organelle morphology. All data were expressed in the form of mean ± standard deviation, ^∗^*P* < 0.05, ^∗∗^*P* < 0.01, and ^∗∗∗^*P* < 0.001.

Western blot was used to detect the expression levels of ferroptosis-related genes in lung tissues of septic mice, and the results indicated that the expression levels of ASCL4 and ATF4 in the CLP group were significantly higher than those in the control group, while SLC7A11 and GPX4 were lower in the CLP group (Figures 2(a)–2(e)), suggesting occurrence of ferroptosis in the lungs of septic mice. By detecting the levels of redox products in lung tissues, we found that the levels of MDA and MPO in the CLP group were higher than those in the control group, while the levels of GSH and SOD were lower in the CLP group (Figures 2(f)–2(i)).

### 3.3. LPS Stimulated Alveolar Epithelial Cells to Trigger Ferroptosis

In cell experiments, we used mouse alveolar epithelial (MLE-12) cells to simulate the sepsis model in vitro via LPS stimulation. We first set 3 different concentrations of LPS (2.5, 5, 10 *μ*g/mL) to treat the cells. Western blot was conducted to detect expression levels of ferroptosis-related genes, and results showed that different concentrations of LPS could increase the expression levels of ASCL4 and ATF4, inhibit the expression levels of SLC7A11 and GPX4, and trigger ferroptosis. 5 *μ*g/mL LPS had the best activation effect on ferroptosis (Figures 2(j)–2(n)). Then, we set three time points (12, 24, and 48 h). Western blot was used to detect the expression levels of related genes, it was found that LPS could trigger ferroptosis at different time points, and the optimal time was 24 h (Figures 2(o)–2(s)). Lipid peroxidation levels were evaluated using a lipid peroxidation probe, and the results showed that the levels of lipid peroxides in the LPS group was significantly higher than those in the control group (Figures 2(t) and 2(u)). In addition, we further verified our conclusions by observing the morphology of cells using electron microscopy. The results showed that, compared with the control group, the membrane of mitochondria in the sepsis group was broken and vacuolated, mitochondrial spine was decreased or absent, and the membrane density obviously increased (Figure 2(v)).

### 3.4. Fer-1 Inhibited the Occurrence of Ferroptosis in Lung Tissues and Alveolar Epithelial Cells of Sepsis

**Figure 3:**
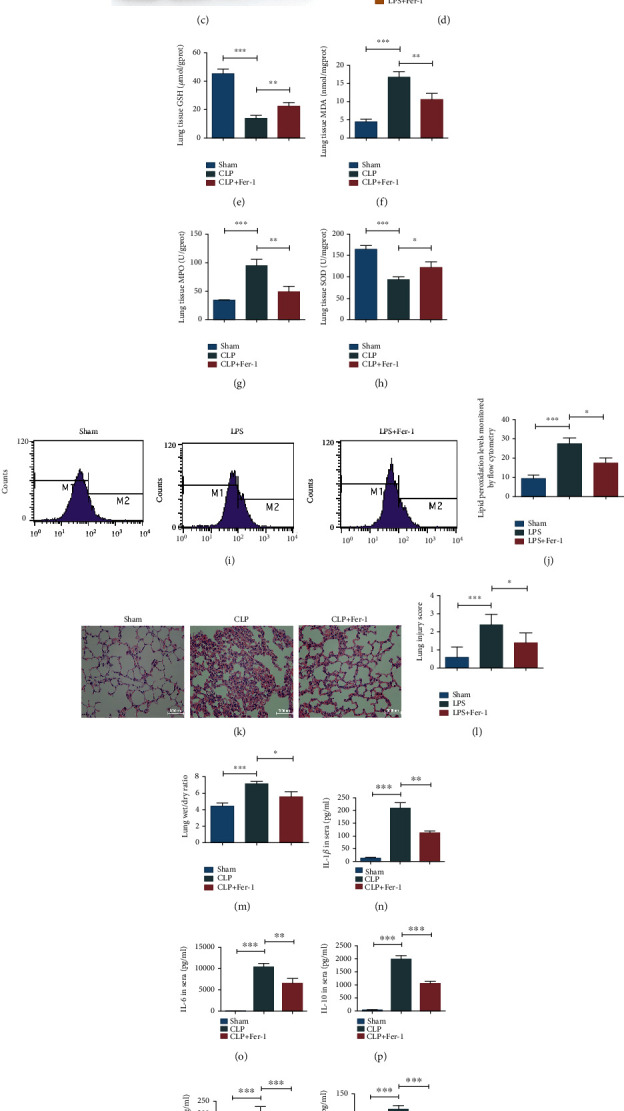
Fer-1 inhibited the occurrence of ferroptosis and alleviated lung injury in lung tissues and alveolar epithelial cells of sepsis. (a, b) Western blot assay was performed to evaluate the effect of Fer-1 on the expression level of GPX4 in lung tissues of septic mice. (c, d) Western blot assay was performed to evaluate the effect of Fer-1 on GPX4 expression in MLE-12 cells. (e–h) Effects of Fer-1 on the expression level of redox products in lung tissues of septic mice. (i, j) Flow cytometry was used to detect the effects of Fer-1 on lipid peroxides in MLE-12 cells. (k) HE staining results in each group. (l) Lung injury score in each group. (m) Wet-dry weight ratio of lung tissues in each group. (n–q) Levels of TNF-*α*, IL-1*β*, IL-6, and IL-10 in sera of each group. (r–u) Levels of TNF-*α*, IL-1*β*, IL-6, and IL-10 in BALF of each group. All data were expressed in the form of mean ± standard deviation; ^∗^*P* < 0.05, ^∗∗^*P* < 0.01, and ^∗∗∗^*P* < 0.001.

Fer-1, a ferroptosis inhibitor, was used to further confirm the occurrence of ferroptosis in septic mice. Fer-1 was intraperitoneally injected, and changes of related indicators in lung tissues were detected 24 hours after modeling. Western blot experiments showed that Fer-1 could significantly reverse the decrease of the GPX4 expression level caused by sepsis and inhibit the occurrence of ferroptosis (Figures 3(a) and 3(b)). In vitro experiments further verified our conclusion (Figures 3(c) and 3(d)). By detecting the levels of redox products in lung tissues, it was found that Fer-1 could increase the levels of GSH and SOD and reduce the levels of MDA and MPO compared with the CLP group (Figures 3(e)–3(h)). Lipid peroxidation levels in MLE-12 cells of the Fer-1 group were significantly lower than those of the LPS group (Figures 3(i) and 3(j)).

### 3.5. Fer-1 Could Alleviate ALI in Sepsis

We explored the effects of Fer-1 on ALI in septic mice. The results showed that Fer-1 could significantly alleviate the severity of acute lung injury caused by sepsis, reduce the aggregation of inflammatory cells in lung tissues, mitigate alveolar injury and edema, and decrease the wet-dry weight ratio (Figures 3(k)–3(m)). We detected the expression levels of TNF-*α*, IL-1*β*, IL-6, and IL-10 in sera and BALF of septic mice using ELISA, and the results indicated that Fer-1 reduced the upregulation of the expression levels of inflammatory factors caused by sepsis in mice (*P* < 0.05) (Figures 3(n) and 3(u)).

### 3.6. Inhibiting MUC1 Could Further Trigger Ferroptosis in Lung Tissues and Alveolar Epithelial Cells of Sepsis

**Figure 4:**
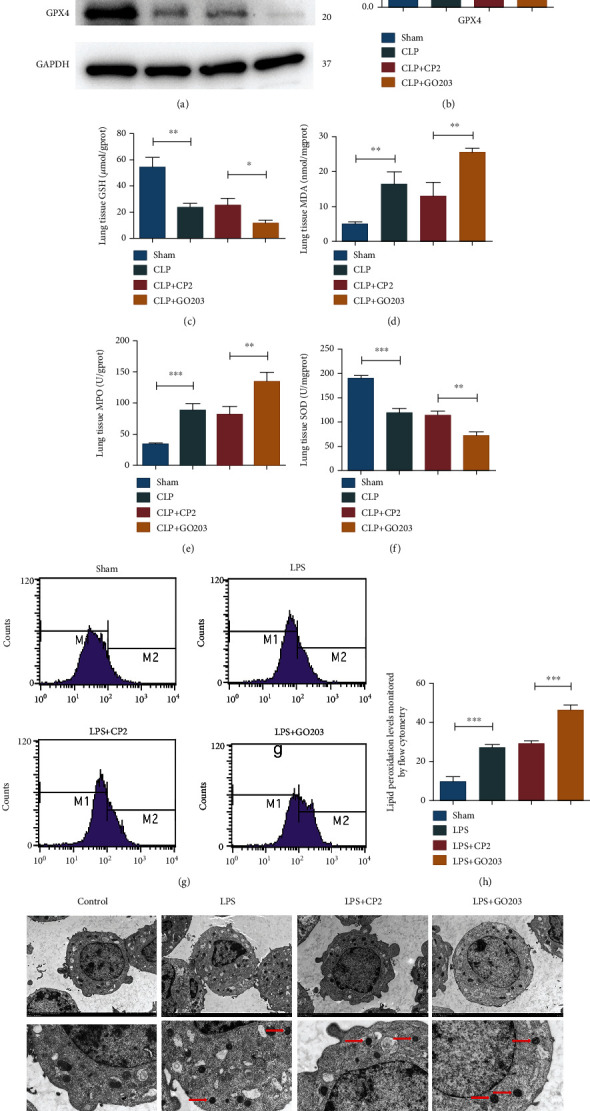
Inhibiting MUC1 triggered ferroptosis in lung tissues and alveolar epithelial cells of sepsis. (a, b) Western blot assay was conducted to evaluate the effect of MUC1 inhibitor GO203 on GPX4 expression in lung tissues of septic mice. (c–f) The expression levels of redox products in lung tissues of septic mice; (g, h) Flow cytometry was used to detect the levels of lipid peroxides in MLE-12 cells. (i) TEM evaluation of the effects of GO203 on the morphology of ferroptosis-related organelles in MLE-12 cells. All data were expressed in the form of mean ± standard deviation; ^∗^*P* < 0.05, ^∗∗^*P* < 0.01, and ^∗∗∗^*P* < 0.001.

To clarify whether MUC1 was involved in the occurrence and development of ferroptosis in sepsis ALI, we treated mice with the MUC1 inhibitor GO203 and its control CP2 before CLP modeling and performed western blot and redox product detection 24 h after modeling to explore the effects of MUC1 on ferroptosis in lungs of sepsis mice. Results showed that MUC1 inhibitors could inhibit the expression level of GPX4 (Figures 4(a) and 4(b)), decrease the expression levels of GSH and SOD, and increase the expression levels of MDA and MPO (Figures 4(c)–4(f)). Besides, inhibiting MUC1 could increase the levels of lipid peroxides (Figures 4(g) and 4(h)), break the membrane of mitochondria, decrease the mitochondrial spine, and increase membrane density in MLE-12 cells stimulated by LPS (Figure 4(i)). The above results indicated that the MUC1 inhibitor could further trigger ferroptosis in lung tissues and alveolar epithelial cells of sepsis.

### 3.7. Inhibiting MUC1 Triggered Ferroptosis through the GSK3*β*/Keap1-Nrf2-GPX4 Pathway

**Figure 5:**
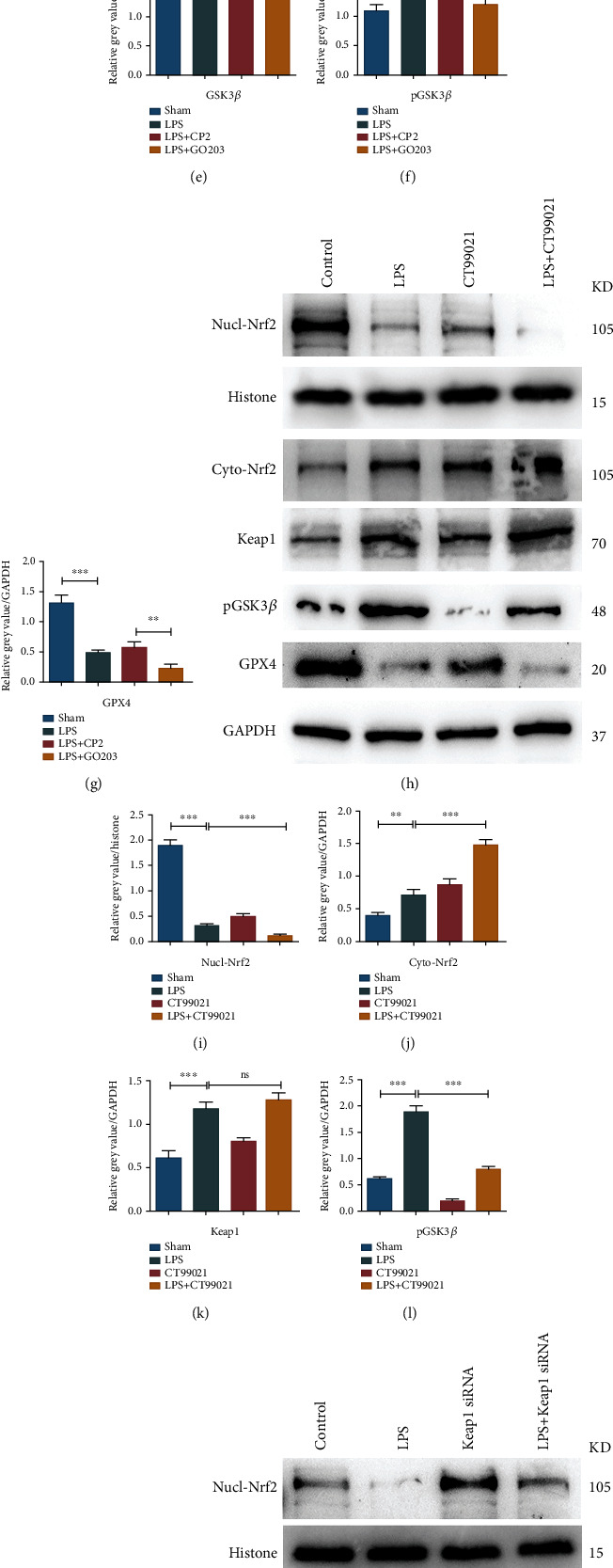
Inhibiting MUC1 triggered ferroptosis via the Keap1/GSK3*β*-Nrf2-GPX4 pathway. (a–g) Effects of inhibiting MUC1 on the expression levels of related genes. (h–m) Effects of GSK3*β* inhibitor CT99021 on related gene expression levels. (n–r) Effects of Keap1 knockdown on the expression levels of related genes. All data were expressed in the form of mean ± standard deviation; ^∗^*P* < 0.05, ^∗∗^*P* < 0.01, and ^∗∗∗^*P* < 0.001.

Western blot results showed that the MUC1 inhibitor could increase the expression level of Keap1, reduce the phosphorylation level of GSK3*β*, inhibit Nrf2 entry into the nucleus, and further reduce the expression level of GPX4 (Figures 5(a)–5(g)). This suggested that MUC1 could activate the expression of GPX4 and inhibit the process of ferroptosis by decreasing Keap1 and improving the phosphorylation level of GSK3*β*, promoting the entry of Nrf2 into the nucleus. To further verify the above results, CT99021, a kind of GSK3*β* inhibitor, was used to treat MLE-12 cells. Western blot results indicated that inhibition of GSK3*β* phosphorylation had no significant effect on Keap1 expression but could significantly increase the accumulation of Nrf2 in the cytoplasm, reduce its entry into the nucleus, and reduce the expression level of GPX4 (Figures 5(h)–5(m)). Besides, Keap1 siRNA was used to inhibit the expression of Keap1, and western blot results showed that inhibiting the expression of Keap1 significantly promoted the accumulation of Nrf2 and improved the expression level of GPX4 (Figures 5(n)–5(r)). The above results suggested that MUC1 could inhibit Keap1 and increase the phosphorylation level of GSK3*β*, thereby promoting the entry of Nrf2 into the nucleus, improving the expression level of GPX4 and inhibiting the ferroptosis process.

### 3.8. Vitamin E Inhibited the Occurrence of Ferroptosis in Lung Tissues and Alveolar Epithelial Cells of Sepsis

**Figure 6:**
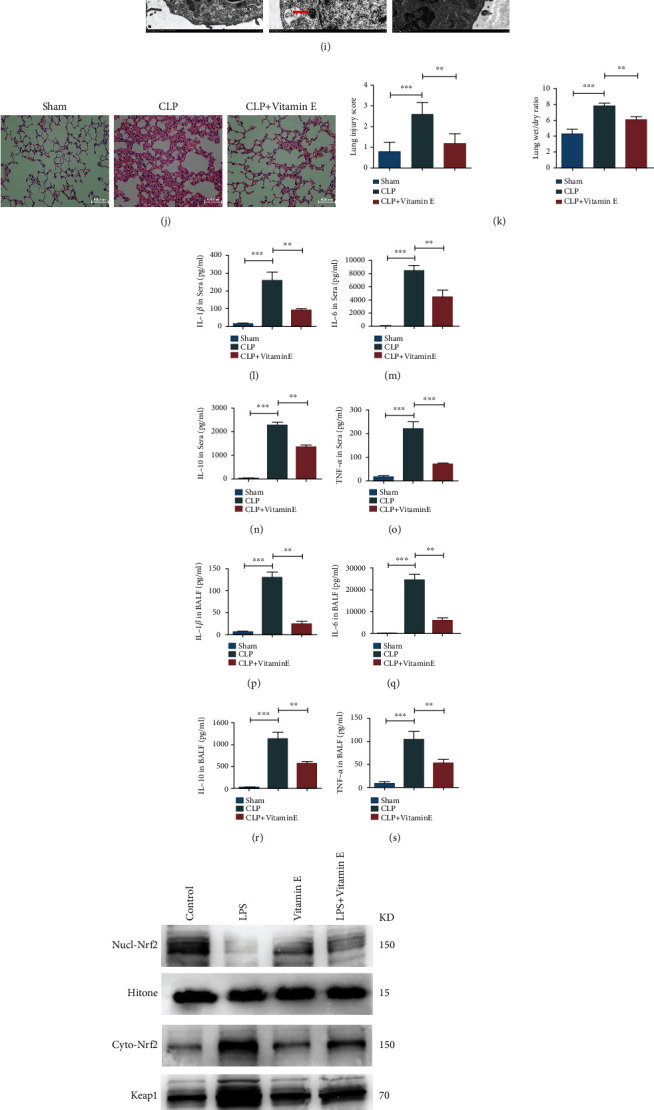
Vitamin E inhibited the occurrence of ferroptosis and alleviated lung injury in lung tissues and alveolar epithelial cells of sepsis. (a, b) Western blot assay was performed to evaluate the effect of vitamin E on the expression level of GPX4 in lung tissues of sepsis mice. (c–f) The expression levels of redox products in lung tissues of sepsis mice. (g, h) Flow cytometry was used to detect the levels of lipid peroxides in MLE-12 cells. (i) The effects of vitamin E on the morphology of ferroptosis-related organelles were assessed by TEM. (j) HE staining results in each group. (k) Lung injury scores and lung wet-dry weight ratio in each group. (l–o) Levels of TNF-*α*, IL-1*β*, IL-6, and IL-10 in sera of each group. (p–s) Levels of TNF-*α*, IL-1*β*, IL-6, and IL-10 in BALF of each group. (t–z) Western blot assay was used to evaluate the effects of vitamin E on the expression levels of related genes. All data were expressed in the form of mean ± standard deviation; ^∗^*P* < 0.05, ^∗∗^*P* < 0.01, and ^∗∗∗^*P* < 0.001.

Vitamin E was intraperitoneally injected after the septic mice were successfully modeled, and the changes of related indicators in lung tissues were detected 24 hours after modeling. Western blot results showed that vitamin E could significantly reverse the decrease of the GPX4 expression level caused by sepsis and inhibit the occurrence of ferroptosis (Figures 6(a) and 6(b)). By detecting the levels of redox products in lung tissues, we found that compared with the sepsis group, vitamin E increased the levels of GSH and SOD and reduced the levels of MDA and MPO (Figures 6(c)–6(f)). Lipid peroxidation levels of MLE-12 cells were evaluated with a lipid peroxidation probe, and the results showed that the lipid peroxide levels in the vitamin E group were lower than those in the LPS group (Figures 6(g) and 6(h)). In addition, we verified our conclusions by observing the morphology of cell ferroptosis via electron microscopy in MLE-12 cells stimulated by LPS. Results indicated that compared with the vitamin E group, the membrane of mitochondria in the LPS group was broken and vacuolated, the mitochondrial spine was decreased or absent, and the membrane density increased (Figure 6(i)).

### 3.9. Vitamin E Alleviated ALI in Sepsis

We explored whether vitamin E had an effect on acute lung injury in septic mice. The results showed that vitamin E alleviated the severity of acute lung injury caused by sepsis, reduced the aggregation of inflammatory cells in the lung tissues, mitigated the alveolar injury and edema, and reduced the wet-dry weight ratio (Figures 6(j) and 6(k)). TNF-*α*, IL-1*β*, IL-6, and IL-10 in sera and BALF of septic mice were detected using ELISA. Results showed that vitamin E reduced the upregulation of inflammatory factors caused by sepsis (*P* < 0.05) (Figures 6(l)–6(s)).

For mechanism studies, western blot analysis showed that vitamin E could inhibit the expression of Keap1 and increase the phosphorylation level of GSK3*β*, reduce the expression level of Nrf2 in the cytoplasm, and promote its entry into the nucleus, thus improving the expression level of GPX4 and inhibiting the process of ferroptosis (Figures 6(t)–6(z)).

### 3.10. MUC1 Had a Sensitizing Effect on Vitamin E

**Figure 7:**
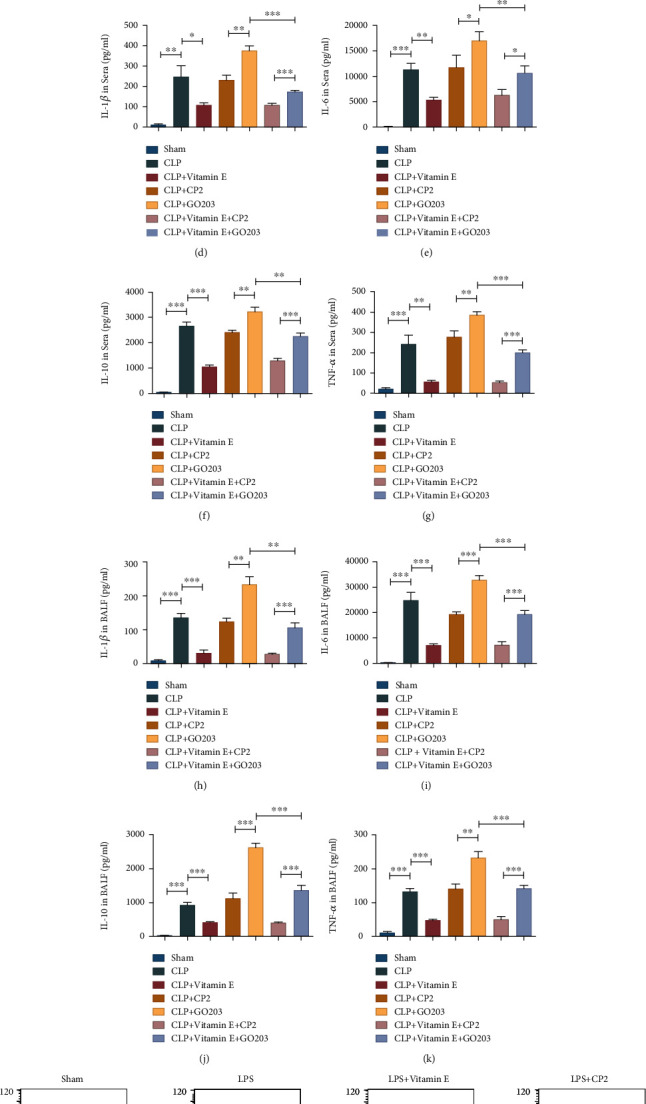
MUC1 sensitized vitamin E to inhibit ferroptosis and relieve lung injury. (a) HE staining results in each group. (b) Lung injury score in each group. (c) Wet-dry weight ratio of lung tissues in each group. (d–g) Levels of TNF-*α*, IL-1*β*, IL-6, and IL-10 in sera of each group. (h–k) Levels of TNF-*α*, IL-1*β*, IL-6, and IL-10 in BALF of each group. (l, m) Flow cytometry was used to detect the levels of lipid peroxides in MLE-12 cells in each group. All data were expressed in the form of mean ± standard deviation; ^∗^*P* < 0.05, ^∗∗^*P* < 0.01, and ^∗∗∗^*P* < 0.001.

**Figure 8:**
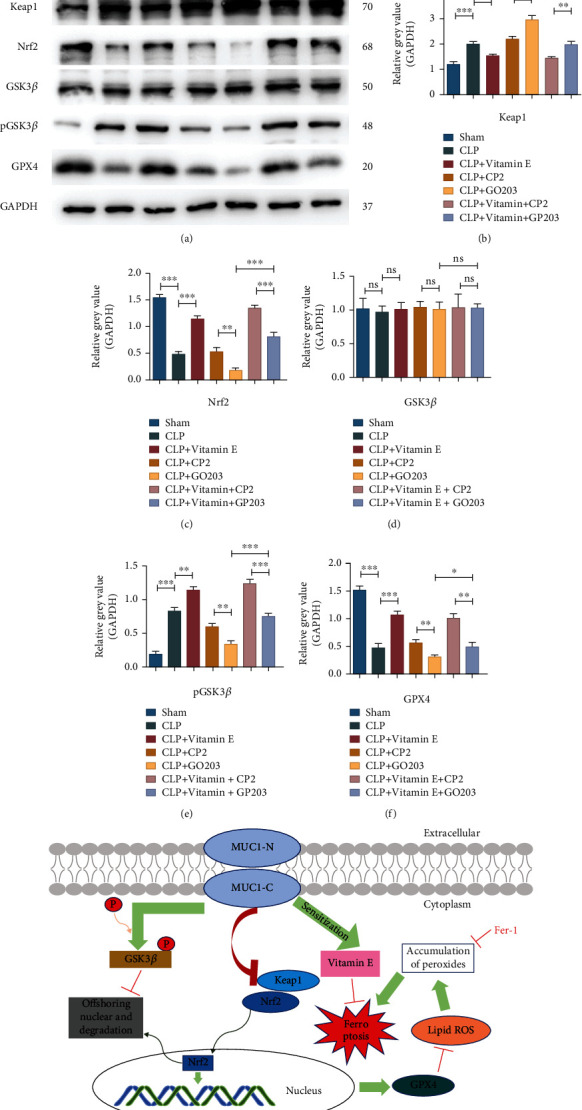
MUC1 sensitized vitamin E to inhibit ferroptosis via the Keap1/GSK3*β*-Nrf2-GPX4 pathway. (a–f) The effects of MUC1 inhibitors and vitamin E on the expression levels of related genes in lung tissues of sepsis mice. (g) The pattern. All data were expressed in the form of mean ± standard deviation; ^∗^*P* < 0.05, ^∗∗^*P* < 0.01, and ^∗∗∗^*P* < 0.001.

In order to explore whether MUC1 could sensitize vitamin E, septic mice were treated with the MUC1 inhibitor GO203 and its control CP2 before CLP modeling, and vitamin E was intraperitoneally injected after modeling. Results showed that MUC1 inhibitor reversed the alleviating effect of vitamin E on acute lung injury caused by sepsis, increased the aggregation of inflammatory cells in the lung tissues, aggravated alveolar injury and edema, and increased the wet-dry ratio (Figures 7(a)–7(c)). ELISA results showed that the MUC1 inhibitor reversed the decreased effect of vitamin E on the expression levels of inflammatory factors in sera and BALF of septic mice (*P* < 0.05) (Figures 7(d)–7(k)). Lipid peroxidation levels in MLE-12 cells were further evaluated using lipid peroxidation probes, and the results showed that the MUC1 inhibitor reversed the decrease in lipid reactive oxygen levels induced via vitamin E (Figures 7(l)–7(m)). Western blot results indicated that the addition of the MUC1 inhibitor reversed the inhibition of Keap1 via vitamin E, reduced the phosphorylation level of GSK3*β*, reduced Nrf2, and finally decreased the expression level of GPX4 (Figures 8(a)–8(f)).

## 4. Discussion

Ferroptosis is a new form of nonapoptotic programmed cell death, which is characterized by iron dependence and accumulation of lipid peroxides [20, 43].

Iron is a kind of redox-active metal and participates in lipid peroxidation and the formation of free radicals [44]. Trivalent iron is reduced to divalent iron in the endosome by iron reductase, which is released into the cytoplasm of an unstable iron pool. Excess trivalent iron is stored in ferritin, and iron can be released into the unstable iron pool when ferritin is depleted, which leads to increased sensitivity to ferroptosis. It has been shown that the dependent lipoxygenase triggers ferroptosis by causing the production of lipid peroxides, and divalent iron can transfer lipid peroxides, leading to extensive lipid peroxidation reactions [38, 45]. The iron metabolism disorder in ARDS patients is closely related to lung tissue injury. Clinical studies have confirmed that the severity of ARDS was related to iron-related proteins [46]. Other researchers detected the levels of total iron and iron regulatory factors in BALF of ARDS patients and found significant changes [47]. Our results indicated that the ferritin level in patients with sepsis or septic shock was significantly higher than that in the control group, which was consistent with the previous results, because ferritin was an inflammatory protein (acute-phase reactant). In addition, we found that sera transferrin and transferrin saturation in patients with sepsis or septic shock were lower than those in the control group, which might be due to the decreased production capacity under stress, as well as the damage to transferrin caused by the increased ability of ferritin to bind iron [48–50]. These results lead to the rapid onset of hypomagnesemia, resulting in obvious lower serum iron levels in patients with sepsis or septic shock. Animal experiments verified our conclusions that the levels of total iron and divalent iron in sera and BALF of sepsis mice were lower than those in the control group, but the levels of total iron and divalent iron in lung tissues were significantly higher than those in the control group, suggesting that there was iron metabolism disorder in the lung of septic mice.

Multiple studies have suggested that ferroptosis played an important role in organ damage caused by sepsis, especially in the lung. It has been reported that ferroptosis inhibitors could significantly improve the prognosis of sepsis, but the specific mechanism remained unclear [25]. In the mouse ALI model, ferroptosis activators could aggravate alveolar inflammation and pulmonary edema and increase the level of inflammatory factors, while these effects could be reversed by ferroptosis inhibitors [26, 27]. We found that expression levels of genes promoting ferroptosis increased significantly in sepsis; however, genes that restrained the ferroptosis were reduced. Redox product levels were changed obviously, and the levels of lipid peroxides increased. Results of the transmission electron microscopy showed that the mitochondrial membrane was broken and vacuolated, and membrane density increased in the mouse alveolar epithelial cells stimulated by LPS. The above results revealed the occurrence of ferroptosis in the lung of sepsis, and the reversal of ferroptosis inhibitors on the above experimental results further confirmed our conclusions.

In recent years, the mechanism of ferroptosis has been partially elucidated; in short, the balance between production and degradation of intracellular lipid peroxides is broken, the antioxidant capacity of cells is reduced, and the accumulation of lipid peroxides is continuous [43]. There are many inducers of ferroptosis, which involve different signaling pathways, but all upstream signaling pathways ultimately reduce the antioxidant capacity of cells by directly or indirectly affecting the activity of glutathione peroxidase (GPX), leading to ferroptosis [51–53]. GPX4, as one of the most important members of the GPX family, plays a crucial role in ferroptosis which can convert glutathione to oxidized glutathione and reduce lipid peroxides to corresponding alcohols. Inhibition of GPX4 leads to accumulation of lipid peroxides, resulting in ferroptosis [54]. Previous studies have confirmed the key regulatory role of GPX4 in the occurrence of ferroptosis [55–57]. Our study found that the expression level of GPX4 in the lung tissues of septic mice and the alveolar epithelial cells stimulated by LPS were significantly lower than those of the control group.

MUC1, as a polymeric transmembrane glycoprotein, plays an important role in many inflammatory diseases, especially respiratory diseases [32]. Our research group has been focused on the role of MUC1 in sepsis-induced ALI/ARDS. Through a series of experiments, we found that MUC1 was involved in the process of paclitaxel-alleviating ALI in septic mice [33], and inhibition of MUC1 dimerization could significantly reduce the severity of ALI and the levels of inflammatory factors in sera and BALF of septic mice. Besides, MUC1 might be a biomarker for predicting whether patients with early sepsis would develop into ARDS, which had important potential application value [34]. However, the mechanism of MUC1 in alleviating lung injury of sepsis was still unclear. In recent years, studies have shown that MUC1 was closely associated with ferroptosis and might be a biomarker related to ferroptosis [35, 36]. Our studies indicated that inhibiting the dimerization of MUC1 could significantly reduce the expression level of GPX4, decrease the expression levels of GSH and SOD, improve MDA and MPO, increase the level of lipid peroxides, break mitochondrial membrane, and increase membrane density, thereby stimulating ferroptosis and aggravating lung injury, which proved the close relationship between MUC1 and ferroptosis in the ALI model of sepsis.

Nuclear erythroid 2 related factor 2 (Nrf2) is an important transcription factor that regulates genes related to iron metabolism during oxidative stress [58, 59], the activation of which can promote iron storage, reduce iron uptake, and limit reactive oxygen species production. Nrf2 has many target genes, among which glutathione peroxidase (GPX) is the most important one. This indicates the close relationship between Nrf2 and ferroptosis. Nrf2 expression is affected by multiple pathways, including Keap1- (Kelch-like-Ech-associated protein 1-) dependent and independent pathways. Keap1 is a high molecular protein anchored to actin [60] and could bind to Nrf2 under normal conditions, which is continuously inactivated through the ubiquitin proteasome pathway. When cells are exposed to oxidative stress or stimulated by cytotoxic agents, Nrf2 dissociates from Keap1 and is incorporated into the nucleus to regulate redox homeostasis of cells [60]. The Keap1-independent pathway is the posttranslational modification of Nrf2, which contains a lot of serine, threonine, and tyrosine residues that provide phosphorylation sites for different kinases (such as GSK3*β*, ERK, PI3K-AKT, and MAPK) [61], leading to nuclear outward migration and degradation of Nrf2. GSK3*β* is a subtype of glycogen synthase kinase 3 [62], which has been proven to be a key factor regulating the stability of Nrf2 and a common downstream effector of many Nrf2 inducers by multiple studies [61, 63]. GSK3*β* stabilizes Nrf2 by phosphorylation of the Neh6 region, which in turn promotes ubiquitination of connexin *β*-TrCP to form a complete E3 ligase, ultimately leading to Nrf2 degradation [64]. GSK3*β* is the downstream targets of AKT, MAPK, and other kinase cascade reactions, and activation of these pathways inhibits GSK3*β* through multiple phosphorylation sites [63], thus helping Nrf2 to play a better role. In our studies, we found that MUC1 inhibitors could increase the expression level of Keap1, reduce the phosphorylation level of GSK3*β*, inhibit the entry of Nrf2 into the nucleus, and reduce the expression level of GPX4. The above conclusions were further verified by the application of GSK3*β* inhibitors and Keap1 siRNA. These results suggested that MUC1 might inhibit the occurrence and development of ferroptosis through the GSK3*β*/KEAP1-NRF2-GPX4 signaling pathway, thus alleviating sepsis-induced ALI.

Vitamin E is a common antioxidant, which has been reported to play an important role in the occurrence and development of ferroptosis in recent years [40]. Qian et al. reported that GPX4 and vitamin E could cooperatively protect hematopoietic stem and progenitor cells from lipid peroxidation and ferroptosis [37]. Another study revealed that vitamin E exerted neuroprotective effects in pentylenetetrazole kindling epilepsy via suppression of ferroptosis [65]. However, whether it is involved in the ferroptosis process of ALI/ARDS in sepsis is still unclear. Our results confirmed the important role of vitamin E in ferroptosis of sepsis-induced ALI and found it could obviously increase the expression levels of genes that inhibited ferroptosis and decrease the expression level of genes that accelerate ferroptosis, change redox product levels, reduce the levels of lipid peroxide levels, and reverse mitochondrial membrane destruction and membrane density increase caused by ferroptosis. Through further exploration, we found that MUC1 had a sensitization effect on vitamin E. MUC1 could promote the protective effect of vitamin E on lungs of sepsis and reduce the levels of inflammatory factors in sera and BALF. In terms of mechanism, MUC1 was able to enhance the inhibitory effect of vitamin E on Keap1 and stimulate the phosphorylation level of GSK3*β*, thereby promoting Nrf2 entry into the nucleus, increasing GPX4 expression, inhibiting ferroptosis process, and finally alleviating acute lung injury in sepsis.

## 5. Conclusion

This study was the first to explore the changes of iron metabolism indicators in ALI/ARDS of sepsis, clarify the importance of ferroptosis in the occurrence and development of ALI/ARDS of sepsis, and reveal the role and specific mechanism of MUC1 in regulating ferroptosis, as well as the sensitization of vitamin E. MUC1 can inhibit Keap1, increase the phosphorylation level of GSK3*β*, and promote Nrf2 entry into the nucleus, thus improving the expression level of GPX4, sensitizing vitamin E, inhibiting ferroptosis, and alleviating acute lung injury in sepsis. These results will provide new ideas for the treatment strategy of ALI/ARDS in sepsis, with important potential application value.

## Data Availability

The data and materials could be acquired from the corresponding authors by reasonable request.

## Abbreviations

MUC1: Mucin 1
ALI: Acute lung injury
ARDS: Acute respiratory distress syndrome
ROS: Reactive oxygen species
DAMPs: Damage-associated molecular patterns
15-LO: 15-Lipooxygenase
Fer-1: Ferrostatin-1
CLP: Cecal ligation and puncture
BALF: Bronchoalveolar lavage fluid
MLE-12: Murine lung epithelial cell line-12
HE: Hematoxylin and eosin
ELISA: Enzyme-linked immunosorbent assay
qRT-PCR: Quantitative real-time polymerase chain reaction
PVDF: Polyvinylidene fluoride
GSH: Glutathione
MDA: Malondialdehyde
MPO: Myeloperoxidase
SOD: Superoxide dismutase
SD: Standard deviation
FBS: Fetal bovine serum
GAPDH: Glyceraldehyde-3-phosphate dehydrogenase
LPS: Lipopolysaccharides
GPX: Glutathione peroxidase
Nrf2: Nuclear erythroid 2 related factor 2
Keap1: Kelch-like-Ech-associated protein 1.

## Supplementary Materials

Supplementary Materials Supplementary Table 1: sequences of primers used for qPCR in this study. Supplementary Table 2: antibodies used for western blotting in this study.
