# Supplementary material for: Mucin 1 Inhibits Ferroptosis and Sensitizes Vitamin E to Alleviate Sepsis-Induced Acute Lung Injury through GSK3β/Keap1-Nrf2-GPX4 Pathway
Source: Oxid Med Cell Longev. 2022 Jul 21;2022:2405943. doi: 10.1155/2022/2405943 (PMC9334047; doi:10.1155/2022/2405943)
Supplement: Supplementary Materials — Supplementary Table 1: sequences of primers used for qPCR in this study. Supplementary Table 2: antibodies used for western blotting in this study. [file 2405943.f1.docx]

**Supplementary Table 1** Sequences of primers used for qPCR in this study

| BMP6-F | CCTGTCCAATGACGACGAAGA |
| --- | --- |
| BMP6-R | TGTGGTGTCGTTGATGTGGG |
| Hamp1-F | AGGGCAGACATTGCGATACC |
| Hamp1-R | GCAACAGATACCACACTGGGA |
| Tfr1-F | CCAGCAACAGATGAGACAGACTAC |
| Tfr1-R | TTCACAACAGATACCACAGGAGG |
| Fth1-F | ATAAAGAAACCAGACCGTGATGACT |
| Fth1-R | GGATTTCACCTGTTCACTCAGATAA |
| Ftl-F | TGCCTCTTATGGACCCTTCTAC |
| Ftl-R | ACAGTCGCCATCAGGATAGTT |
| Fpn-F | TGGTGACTGGGTGGATAAGAATG |
| Fpn-R | TCAGGATGTAGCAGACAGTAAGGAC |
| GAPDH-F | CCTCGTCCCGTAGACAAAATG |
| GAPDH-R | TGAGGTCAATGAAGGGGTCGT |

**Supplementary Table 2** Antibodies used for western blotting in this study

|  | | | | |
| --- | --- | --- | --- | --- |
| **Antibody** | **Company** | **Cat. No.** | **Source** | **Dilution** |
| ASCL4 | Proteintech | 22401-1-AP | Rabbit | 1:1000 |
| SLC7A11 | Proteintech | 26864-1-AP | Rabbit | 1:1000 |
| ATF4 | Proteintech | 10835-1-AP | Rabbit | 1:1000 |
| GPX4 | Signalway Antibody | 49731-1 | Rabbit | 1:1000 |
| Nrf2 | Proteintech | 16396-1-AP | Rabbit | 1:1000 |
| Keap1 | Proteintech | 10503-2-AP | Rabbit | 1:1000 |
| GSK3β | Proteintech | 22104-1-AP | Rabbit | 1:1000 |
| pGSK3β | Proteintech | 14850-1-AP | Rabbit | 1:1000 |
| Histone H3 | Abcam | 1791 | Rabbit | 1:1000 |
| GAPDH | Cell signaling | 5174 | Rabbit | 1:1000 |
